# Biochemical Mechanisms of Cellular Stress Adaptation in the Pathogenesis of Chronic Diseases

**DOI:** 10.3390/molecules31091381

**Published:** 2026-04-22

**Authors:** Joanna Lemanowicz, Sylwester M. Kloska, Anetta Siwik-Ziomek, Paweł Kołaczyk, Urszula Wnuk Lipińska, Anna Kloska

**Affiliations:** Division of Biochemistry, Faculty of Medicine, Bydgoszcz University of Science and Technology, 85-796 Bydgoszcz, Poland; sylwester.kloska@pbs.edu.pl (S.M.K.); anetta.siwik-ziomek@pbs.edu.pl (A.S.-Z.); pawel.kolaczyk@pbs.edu.pl (P.K.); urstoj000@pbs.edu.pl (U.W.L.); anna.kloska@pbs.edu.pl (A.K.)

**Keywords:** chronic disease, endoplasmic reticulum, lifestyle-related diseases, mitochondria, non-communicable disease, oxidative stress, reactive oxygen species, stress response

## Abstract

Chronic diseases increasingly reflect a shared biological origin: persistent cellular stress. This review summarizes the biochemical mechanisms that normally preserve cellular homeostasis, namely redox regulation, endoplasmic reticulum proteostasis, mitochondrial quality control, autophagy, and DNA damage response, and explains how they fail under sustained lifestyle-related overload. Repeated exposure to psychological stress, sleep disruption, hypercaloric intake, and physical inactivity shifts adaptive signaling toward maladaptation, promoting oxidative damage, protein misfolding, mitochondrial dysfunction, low-grade inflammation, and genomic instability. These interconnected processes contribute to the development and progression of major chronic non-communicable diseases, including obesity, type 2 diabetes, cardiovascular disease, neurodegeneration, and cancer. Particular emphasis is placed on circadian and neuroendocrine regulation, especially overactivation of the hypothalamic–pituitary–adrenal axis and impaired nocturnal regenerative pathways such as glymphatic clearance and DNA repair. Together, the evidence supports a unifying model in which chronic pathology emerges from cumulative failure of cellular resilience systems rather than isolated organ-specific defects. This perspective highlights sleep optimization, stress reduction, and metabolic regulation as mechanistically grounded strategies for prevention and supportive interventions for chronic disease.

## 1. Introduction

The term “stress” is commonly associated with psychological strain, time pressure, sleep deprivation, and the persistent demands of modern life. It is typically framed as an emotional or behavioral phenomenon. However, the most fundamental dimension of stress is biological and unfolds at the level of individual cells.

Every cell in the human body operates within an environment of continuous biochemical challenge. It must sustain ATP production, synthesize and properly fold thousands of proteins, preserve genomic integrity, and dynamically adjust to fluctuations in nutrient and oxygen availability. These processes are executed with remarkable precision, yet they are intrinsically error-prone. It is estimated that tens of thousands of DNA lesions arise daily in a single human cell [1], while a substantial proportion of newly synthesized polypeptides require refolding or proteasomal degradation before achieving their native conformation [2]. Collectively, these observations indicate that cellular homeostasis depends on tightly coordinated surveillance and repair networks.

In biological terms, cellular stress refers to conditions in which the capacity to maintain homeostasis is perturbed. Importantly, cellular stress is not inherently pathological. Transient and moderate deviations from equilibrium function as adaptive stimuli, activating cytoprotective pathways that enhance stress resistance and promote cellular resilience [3]. Such adaptive responses are essential for maintaining organismal integrity in fluctuating environments.

Pathology emerges when stress becomes chronic rather than episodic. Under sustained overload, cellular repair and quality control systems progressively lose efficiency. Misfolded proteins accumulate within the endoplasmic reticulum (ER), mitochondrial bioenergetic performance declines, and excessive production of reactive oxygen species (ROS) leads to oxidative damage of lipids, proteins, and nucleic acids [4]. At the organismal level, these processes converge as shared molecular denominators of numerous non-communicable chronic diseases.

Growing evidence indicates that the boundary between psychological stress and cellular stress is mechanistically permeable. Chronic emotional strain, sleep disruption, and physical inactivity influence endocrine signaling, inflammatory tone, and systemic metabolism, thereby directly modulating intracellular biochemical networks. Lifestyle factors, often categorized in behavioral terms, thus exert measurable effects on redox balance, proteostasis, mitochondrial function, and genomic stability.

This review examines stress as a biological process embedded in cellular physiology, outlines the principal molecular stress-response pathways, and evaluates how persistent pathway activation contributes to chronic disease pathogenesis. Furthermore, we discuss how diet, sleep, and physical activity influence adaptive capacity at the cellular level, framing lifestyle modification as a form of molecular-level prevention.

## 2. Origins of Cellular Stress

**Figure 1:**
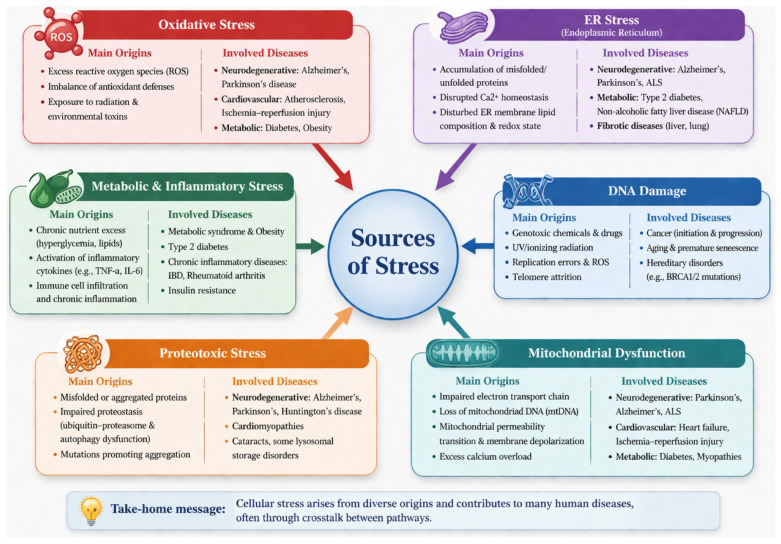
Sources of stress.

Cellular stress may originate from both exogenous factors, such as toxins, oxygen deprivation, or radiation, and endogenous factors resulting from high metabolic activity, excessive ROS production, or overload of the protein synthesis machinery [5,6,7]. Disruption of intracellular homeostasis consequently triggers the activation of adaptive cellular programs or, alternatively, the initiation of regulated cell death pathways [8,9]. Numerous studies have demonstrated that the principal sources of cellular stress include oxidative stress, ER stress, DNA damage, hypoxia, mitochondrial dysfunction, proteotoxic stress, as well as metabolic and inflammatory stress [10,11,12] (Figure 1).

Oxidative stress represents a disturbance of cellular redox homeostasis characterized by excessive ROS production [13]. ROS comprise a group of highly reactive molecules, including both oxygen-centered free radicals and non-radical species such as hydrogen peroxide (H_2_O_2_) [14]. Under physiological conditions, ROS serve essential functions in intracellular signaling and participate in innate and adaptive immune responses. However, disruption of redox balance results in oxidative modification of biomolecules, including proteins, lipids, and DNA, leading to dysregulation of cellular signaling pathways. This process constitutes a critical mechanistic component of carcinogenesis, aging, and regulated cell death.

In addition to ROS, reactive nitrogen species (RNS) represent a major class of redox-active molecules that contribute to both intracellular signaling and nitrosative stress under pathological conditions. RNS constitute an important complementary component of cellular redox biology. They are generated primarily from nitric oxide (NO), which is synthesized by nitric oxide synthases (NOS), and include highly reactive derivatives such as peroxynitrite (ONOO^−^), nitrogen dioxide (NO_2_·), and dinitrogen trioxide (N_2_O_3_). Under physiological conditions, NO plays essential roles in vasodilation, neurotransmission, mitochondrial regulation, and immune defense. However, excessive NO production, particularly through inducible nitric oxide synthase (iNOS) during chronic inflammation, promotes the formation of secondary RNS through reactions with superoxide anion (O_2_$\cdot^{-}$), leading to peroxynitrite generation. Peroxynitrite is a potent oxidizing and nitrating agent capable of modifying proteins, lipids, and nucleic acids, thereby disrupting enzyme activity, membrane integrity, and genomic stability. Persistent nitrosative stress has been implicated in endothelial dysfunction, insulin resistance, neurodegeneration, and carcinogenesis. Cellular defense against RNS involves both enzymatic and non-enzymatic systems, including superoxide dismutase, glutathione-dependent pathways, peroxiredoxins, and thioredoxin systems, which collectively limit nitrative damage and maintain redox homeostasis.

Cell death can manifest in multiple forms, including apoptosis, necroptosis, oxeiptosis, pyroptosis, and parthanatos, each distinguished by specific genetic determinants, biochemical features, and signaling cascades [15].

ROS are generated predominantly in mitochondria as by-products of oxidative phosphorylation during cellular respiration. They may be conceptualized as molecular “sparks” produced by the mitochondrial respiratory machinery. When the bioenergetic system becomes overloaded or dysfunctional, ROS generation increases substantially, promoting oxidative modification of cellular macromolecules and thereby initiating oxidative stress [16].

One of the less immediately apparent yet critically important sources of cellular stress is the ER, an organelle that functions as a central hub for protein synthesis, folding, and quality control. Within the ER lumen, newly synthesized polypeptides undergo precise conformational maturation to achieve their functional three-dimensional structure [17].

When intracellular conditions deteriorate, such as during nutrient excess, inflammatory signaling, or hypoxia, the efficiency of protein folding becomes compromised. If the burden of misfolded or unfolded proteins exceeds the corrective capacity of the ER quality control system, a conserved signaling cascade known as the unfolded protein response (UPR) is activated [18,19]. Misfolded proteins accumulate within the ER lumen, effectively disrupting proteostasis. Under physiological conditions, ER-resident chaperones and degradation pathways attempt to refold or eliminate aberrant proteins before they exert cytotoxic effects [20].

The primary objective of the UPR is to restore homeostasis by transiently attenuating global protein synthesis, upregulating molecular chaperones, and enhancing ER-associated degradation (ERAD) pathways. However, when ER stress becomes chronic, this adaptive response transitions into a maladaptive program. Sustained UPR signaling promotes pro-inflammatory pathways and the activation of cell death mechanisms [21]. At this critical juncture, a response initially designed to preserve cellular integrity becomes a contributing factor to disease pathogenesis.

DNA damage constitutes another major source of cellular stress. The DNA damage response (DDR) encompasses a coordinated network of signaling pathways activated upon detection of genomic lesions. Activation of the DDR leads to cell cycle arrest, facilitating DNA repair processes, or alternatively promotes apoptosis or cellular senescence when damage is irreparable [22].

Cellular senescence represents a stable state of irreversible cell cycle arrest triggered by diverse stressors, including DNA damage, redox imbalance, and oncogenic signaling. Senescent cells lose proliferative potential while retaining metabolic and transcriptional activity, a phenotype orchestrated by canonical pathways such as p53/p21^∧^CIP1^∧^ and p16^∧^INK4a^∧^/Rb. A defining feature of these cells is the senescence-associated secretory phenotype (SASP), which exerts profound paracrine effects by secreting pro-inflammatory cytokines, chemokines, growth factors, and matrix-remodeling proteases, thereby reshaping the tissue microenvironment and influencing systemic homeostasis. Redox regulation and hormetic signaling critically modulate the threshold for senescence induction and dictate the functional outcomes of this program. Emerging therapeutic strategies, notably senolytic interventions, aim to selectively eliminate senescent cells, reconciling their context-dependent protective and deleterious roles, and offering translational avenues for ameliorating age-related pathologies [23,24].

Chronic activation of the DDR, resulting from environmental stressors, persistent oxidative stress, or exposure to chemotherapeutic agents, contributes to the induction of cellular senescence and genomic instability, particularly in malignant cells. The serine/threonine kinases ataxia-telangiectasia mutated (ATM) and ATM- and Rad3-related (ATR), along with their downstream effectors checkpoint kinase 1 (Chk1) and checkpoint kinase 2 (Chk2), represent central regulatory nodes within DDR signaling cascades [25]. These kinases orchestrate DNA repair, enforce cell cycle checkpoints, and modulate apoptotic pathways. Pharmacological inhibition of ATM/ATR signaling has been shown to enhance tumor sensitivity to chemotherapy and radiotherapy, underscoring their therapeutic relevance in oncology.

Mitochondria are frequently referred to as the “powerhouses” of the cell for good reason. It is within these organelles that the majority of the energy required for life is generated and stored in the form of ATP molecules [26]. This process relies on a precisely regulated flow of electrons, enabling the cell to efficiently harvest energy, although not without cost. In the absence of functional mitochondria, cells rapidly lose their capacity to maintain normal physiological activity.

However, the role of mitochondria extends far beyond energy production. They participate in the regulation of ion homeostasis, including calcium signaling; contribute to lipid metabolism; and determine cellular fate by initiating programmed cell death when damage becomes irreversible. Moreover, mitochondria possess their own genetic material (mtDNA), which encodes essential components of the oxidative phosphorylation machinery—an evolutionary remnant of their bacterial ancestry [12,16].

Under conditions of cellular stress, mitochondria cease to function merely as bioenergetic organelles and instead assume the role of a central regulatory hub. They are among the first to respond to alterations in energy supply, oxygen availability, and nutrient status, translating metabolic cues into defined biochemical responses [27]. In this context, mitochondria act as sensors of cellular state, continuously evaluating whether conditions favor adaptation or approach the limits of cellular resilience.

Mitochondrial efficiency is tightly dependent on the balance between substrate availability and cellular energy demand. Under conditions of chronic oversupply of glucose and fatty acids, the electron transport chain becomes highly reduced, leading to an elevated mitochondrial membrane potential [28]. Instead of the efficient four-electron reduction of oxygen to water at the designated catalytic centers of respiratory complexes, electrons may be prematurely transferred directly to molecular oxygen outside these reaction sites. Mitochondrial dysfunction also alters nitric oxide metabolism, thereby promoting secondary nitrosative stress.

The resulting excess of ROS induces secondary damage to mitochondrial DNA, enzymatic proteins, and the lipids composing the inner mitochondrial membrane. Such structural impairments compromise oxidative phosphorylation, paradoxically decreasing bioenergetic efficiency while simultaneously increasing metabolic toxicity [29]. In this context, mitochondria subjected to sustained energetic pressure cease to function as efficient energy generators and instead become major sources of intracellular damage, representing a fundamental mechanism underlying accelerated molecular aging [16].

ATP production in the respiratory chain is inseparably linked to the generation of ROS [30]. In small quantities, ROS serve useful signaling functions; however, when mitochondrial function becomes impaired, the accumulation of these reactive molecules can rapidly escape physiological control. Excess ROS damages proteins, lipids, and DNA, thereby initiating oxidative stress [12,16]. Consequently, cells must not only generate energy but also continuously monitor the functional integrity of their bioenergetic machinery.

Mitochondrial DNA is particularly vulnerable to oxidative damage. It encodes essential components of the energy production apparatus; thus, its structural or functional disruption leads to progressively reduced ATP synthesis accompanied by increased production of harmful metabolites. This establishes a self-reinforcing feedback loop in which declining energetic efficiency intensifies cellular stress, while accumulating stress further impairs mitochondrial function [30].

Moreover, severely damaged mitochondria may release fragments of their DNA into the cytoplasm and extracellular space. From the perspective of the immune system, such molecules function as danger-associated signals analogous to those observed during infectious processes. Cellular sensing of these signals triggers inflammatory pathways and cytokine production, which, at the organismal level, may contribute to the development of chronic low-grade inflammation.

It is therefore not surprising that mitochondrial dysfunction is implicated in numerous chronic non-communicable diseases, ranging from metabolic and cardiovascular disorders to neurodegenerative diseases, autoimmune conditions, and cancer [12,31,32]. Increasing evidence suggests that persistent systemic inflammation, a common feature of these conditions, often originates at the level of cellular bioenergetic dysfunction [33].

Chronic metabolic stress arises when cells are persistently exposed to an excess supply of nutrients, particularly glucose and lipids. Although such conditions may appear energetically favorable, they in fact represent a state of functional overload. Cellular systems responsible for energy processing, detoxification of metabolic by-products, and protein quality control are not designed to operate continuously at maximal capacity and may gradually lose efficiency under sustained pressure [34].

Persistently elevated glucose levels, such as those observed in obesity, high-calorie diets, or insulin resistance, lead to excessive flux of substrates through metabolic pathways. This results in increased ROS production, as discussed previously. These highly reactive molecules can damage proteins, lipids, and cellular structures, thereby promoting oxidative stress [35]. Additionally, glucose excess facilitates the formation of reactive metabolic intermediates that modify protein structures, impairing proper folding and functional activity [36,37].

**Figure 2:**
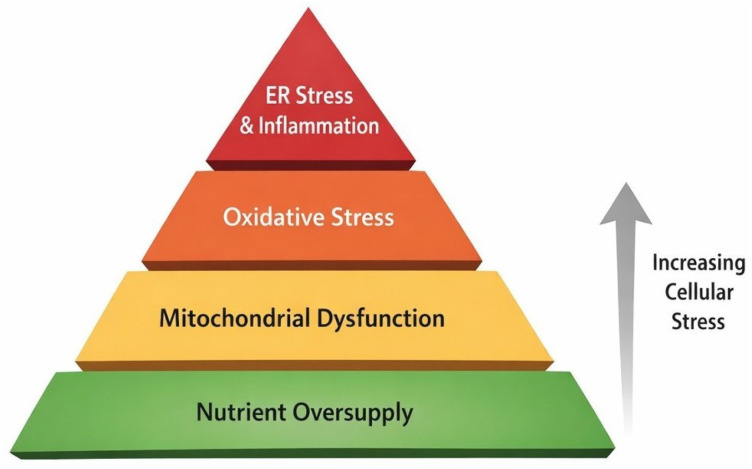
Metabolic stress pyramid.

The metabolic stress pyramid (Figure 2) illustrates the progressively increasing cellular burden resulting from excessive availability of primary energetic substrates such as glucose, fatty acids, and amino acids [38,39]. Pancreatic β-cells are particularly susceptible to such metabolic perturbations. These cells are responsible for insulin synthesis and secretion, and prolonged functional overload leads to dysregulation of gene expression programs essential for their survival and activity. Consequently, β-cells gradually lose their insulin secretory capacity, contributing to the pathogenesis of type 2 diabetes.

From a cellular perspective, metabolic stress should therefore not be interpreted merely as an excess of energy supply but rather as a state of chronic physiological overload that slowly disrupts intracellular homeostasis and promotes the development of chronic non-communicable diseases [40].

Proteotoxic stress refers to the accumulation of misfolded proteins and protein aggregates, a process that disrupts the cellular network responsible for maintaining stress resilience [41]. Such accumulation impairs normal cellular functions and reduces cell viability [42]. The presence of protein aggregates triggers cellular defense responses, including activation of heat-shock pathways and increased demand for proteasomal degradation of damaged proteins [43,44].

When proteotoxic burden becomes severe or chronic, pathological processes tend to outweigh cellular repair mechanisms. Targeted modulation of proteostasis, for example through enhancement of molecular chaperone activity, restoration of autophagic flux, or selective removal of protein aggregates, represents a promising therapeutic strategy. However, such interventions require precise adaptation to the characteristics of the affected tissue and the specific type of protein aggregates involved [44,45].

Growing evidence indicates that chronic inflammation is not a singular pathological entity but rather the result of a coupling of multiple stress sources acting simultaneously within the cell. When homeostatic balance is disrupted, mechanisms originally designed to protect cellular integrity may begin to reinforce one another in a self-amplifying manner.

Excess ROS can damage nuclear and mitochondrial DNA, allowing fragments of genetic material to appear in intracellular or extracellular locations where they are normally absent. The cell interprets such events as danger signals and activates intrinsic alarm pathways that initiate inflammatory responses. A major limitation of this protective reaction is that inflammatory signaling itself can further enhance oxidative stress, thereby trapping the cell in a self-sustaining inflammatory feedback loop.

Concurrently, ER overload disturbs protein folding and metabolic regulation, intensifying pro-inflammatory signaling. Damaged mitochondria may release mitochondrial DNA into the cytoplasm, which functions as a danger-associated molecular pattern that stimulates immune activation [46].

As a result, distinct forms of cellular stress cease to operate independently and instead merge into a single pathological network of interacting disturbances. This interconnected stress system underlies the pathogenesis of many chronic diseases and is currently considered one of the key targets of modern therapeutic research [47,48,49].

## 3. Cellular Stress Response: Adaptation, Biological Cost and Limits of Resilience

The cell is not a passive victim of stress. Any disruption of its internal homeostasis, whether due to an excess of reactive molecules, metabolic overload, or the accumulation of defective proteins, initiates a complex decision-making process aimed at a single objective: survival at the lowest possible biological cost.

Importantly, the cellular response to stress is not binary. The same stimulus may promote adaptive remodeling and enhanced stress resistance under transient or moderate conditions, yet lead to structural damage, inflammation, and ultimately cell death when exposure is prolonged or excessive.

The outcome depends critically on the intensity and duration of the stressor, as well as on the metabolic context in which it occurs. Acute, low-level stress can activate protective pathways that improve resilience and restore homeostasis. In contrast, chronic or severe stress shifts the balance toward maladaptive signaling, bioenergetic failure, and activation of pro-inflammatory or pro-apoptotic cascades. Thus, cellular fate is determined not merely by the presence of stress, but by its quantitative and temporal characteristics within a given physiological environment.

### 3.1. Stress as Information, Not Merely a Threat

Under physiological conditions, stress functions as an early warning signal. A moderate increase in ROS, transient ER overload, or a temporary energy deficit informs the cell that environmental conditions are changing and require adaptation. Such signals initiate adaptive responses, including metabolic reprogramming, reinforcement of quality-control mechanisms, and temporary restriction of energetically demanding processes such as protein synthesis and proliferation [50]. From a biochemical perspective, stress therefore serves as an informational cue: it instructs the cell to modify its functional strategy. The challenge arises when the warning signal fails to subside.

Every defensive response carries a biological cost. Activation of antioxidant enzymes, synthesis of molecular chaperones, induction of autophagy, and DNA repair all require energy, substrates, and time. In the short term, this represents a beneficial investment that enhances cellular survival. However, under chronic stress conditions, the energetic and metabolic costs may outweigh the benefits. Cells compelled to remain in a persistent defensive state operate in an emergency mode: specialized functions are reduced, metabolic profiles are altered, and pro-inflammatory signals are generated. At the level of tissues and organs, this adaptive-to-maladaptive transition contributes to progressive functional decline; at the organismal level, it promotes the development of chronic diseases [51].

### 3.2. Critical Point: When Adaptation Becomes a Pathology

A central concept in stress biology is the adaptive threshold. As long as stress exposure remains transient and moderate, activated protective mechanisms enhance cellular resilience. However, once this threshold is exceeded, the very same signaling pathways begin to exert deleterious effects.

**Figure 3:**
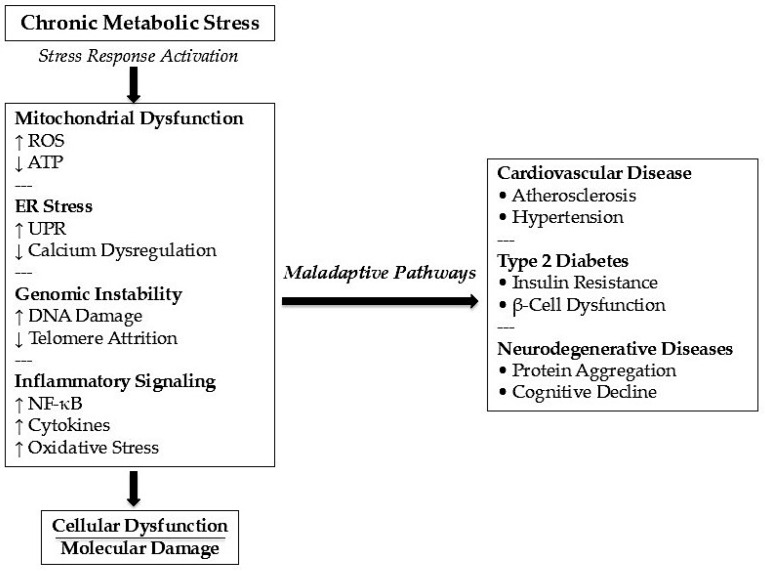
Molecular pathways linking chronic metabolic stress to chronic non-communicable diseases.

Chronic activation of stress responses stabilizes pro-inflammatory signaling, disrupts communication between organelles, and promotes the progressive accumulation of molecular damage. This shift from adaptation to maladaptation explains why evolutionarily conserved defense mechanisms, originally designed to preserve cellular and organismal survival, can become drivers of pathology under sustained activation (Figure 3).

Such mechanisms contribute to the pathogenesis of major chronic diseases, including type 2 diabetes [52], atherosclerosis [53], and neurodegenerative disorders [54].

The cell operates within a dynamic ecosystem in which the boundary between adaptation and damage is fluid and shaped by genetic, metabolic, and environmental determinants. To preserve homeostasis under stress conditions, cells rely on a coordinated network of specialized defense systems [55]. Each system addresses a distinct dimension of cellular crisis: regulation of chemical reactivity, maintenance of protein quality, preservation of mitochondrial integrity, removal of damaged structures, and repair of genetic material.

These protective mechanisms function in an integrated and hierarchical manner. Antioxidant systems limit excessive chemical reactivity and prevent uncontrolled oxidative damage. Protein quality control pathways, including UPR, restore proteostasis under ER stress. Autophagy and selective organelle turnover mechanisms eliminate dysfunctional cellular components, thereby preventing the accumulation of toxic aggregates. In parallel, DNA repair pathways safeguard genomic stability and ensure the continuity of essential cellular functions.

In the following sections, these mechanisms will be examined in greater detail, from antioxidant networks that restrain biochemical instability, through UPR signaling and autophagic processes, to DNA repair systems. Understanding how these pathways cooperate to sustain cellular survival, and why they sometimes fail, is essential for elucidating the molecular basis of chronic disease and age-associated decline.

### 3.3. Antioxidants—Control of Chemical Chaos

One of the earliest and most universal indicators of cellular stress is an increase in intracellular chemical reactivity. ROS and RNS [13] arise naturally as by-products of cellular metabolism—particularly within mitochondria—and perform essential signaling functions under physiological conditions.

The antioxidant defense network in senescent cells involves coordinated activity of regulatory pathways such as Nrf2 signaling and the glyoxalase (Glo) system, including its major isoforms GLO1 and GLO2. Nrf2 acts as a master regulator of redox homeostasis by controlling the expression of antioxidant and cytoprotective genes, thereby modulating oxidative stress responses and senescence-associated processes. In parallel, the glyoxalase system detoxifies reactive dicarbonyls such as methylglyoxal, linking carbonyl stress with redox imbalance, and the establishment of cellular senescence. These pathways are functionally interconnected with metabolic and stress-response networks that sustain the senescent phenotype and influence SASP activity [56,57].

Pathology emerges when reactive species production exceeds the cell’s capacity for regulation. In such circumstances, biochemical order gives way to molecular instability. To prevent the destructive consequences of excessive reactive species, cells have evolved a multilayered antioxidant defense system. Importantly, the objective of this system is not the complete elimination of ROS, but rather the maintenance of their concentrations within a safe and functionally appropriate range [58].

This dynamic balance allows reactive species to retain their signaling roles while minimizing oxidative damage to proteins, lipids, and nucleic acids. The antioxidant network therefore acts not as a suppressor of redox biology, but as a regulatory framework that preserves controlled chemical reactivity within the cellular environment.

A central component of cellular oxidative stress control is glutathione (GSH), a low-molecular-weight tripeptide composed of glutamate, cysteine, and glycine. Owing to the presence of its reactive thiol (-SH) group, glutathione functions as a chemical buffer capable of directly neutralizing ROS and regenerating other antioxidants [59].

The ratio between reduced GSH and its oxidized disulfide form (GSSG) is considered one of the most reliable indicators of cellular redox status [60]. A decline in the GSH/GSSG ratio reflects a shift toward an oxidizing intracellular environment and signals the activation of additional protective mechanisms.

Importantly, the glutathione system is tightly integrated with cellular energy metabolism. Regeneration of GSH from GSSG requires reducing equivalents in the form of NADPH. A major source of NADPH is the pentose phosphate pathway, linking antioxidant defense directly to metabolic flux and substrate availability [61]. Thus, glutathione does not operate in isolation but constitutes a metabolically coupled redox buffering system that integrates oxidative balance with broader bioenergetic and biosynthetic processes.

Beyond low-molecular-weight antioxidants, cells rely on specialized enzymes that act rapidly and selectively to control reactive species. The first line of enzymatic defense is superoxide dismutase (SOD), which catalyzes the conversion of the superoxide anion radical into hydrogen peroxide [62]. Although hydrogen peroxide is less reactive than superoxide, it remains potentially harmful and must be further detoxified.

Hydrogen peroxide is subsequently neutralized either by catalase (CAT) or by glutathione peroxidase (GPx), depending on cellular compartmentalization and metabolic context. This cascade operates in a coordinated manner: SOD limits the accumulation of superoxide, while CAT and GPx prevent hydrogen peroxide from generating highly reactive hydroxyl radicals through Fenton-type reactions. The system resembles a highly organized emergency response network, in which each enzyme fulfills a specific role, and their integration ensures efficient containment of oxidative threats.

Importantly, the activity of these enzymes is genetically regulated and dependent on the availability of essential micronutrients. For example, selenium is required for optimal function of glutathione peroxidases, whereas zinc and copper serve as cofactors for certain SOD isoforms. This interdependence links oxidative stress biochemistry directly to nutritional status and micronutrient balance, underscoring the systemic dimension of cellular redox regulation.

Contrary to common perception, ROS are not merely harmful by-products of metabolism. At moderate concentrations, they function as signaling molecules that regulate key biological processes, including cell proliferation, differentiation, immune responses, and adaptation to physical exercise [63].

ROS participate in redox-sensitive signaling pathways by reversibly modifying specific amino acid residues, particularly cysteine thiols, within regulatory proteins. Through such mechanisms, they modulate kinase activity, transcription factor function, and metabolic flux, thereby integrating environmental cues with intracellular responses.

Complete suppression of ROS signaling could therefore paradoxically impair normal cellular physiology. For this reason, the antioxidant system does not operate as an on–off switch that eliminates reactive species entirely. Rather, it functions as a finely tuned regulatory network that maintains redox signaling within an optimal range—sufficient to preserve physiological communication while preventing oxidative damage.

Knowledge regarding the destructive potential of oxidative stress has contributed to the widespread popularity of antioxidant supplementation. However, clinical studies have shown that the effects of such interventions are not uniformly beneficial [64]. In many cases, high-dose antioxidant supplementation fails to produce the expected health benefits and may even disrupt endogenous adaptive cellular mechanisms [65,66,67].

A notable example is physical exercise, which induces a controlled increase in ROS production that subsequently strengthens endogenous defense systems and improves mitochondrial function [68]. Excessive antioxidant supplementation may attenuate this beneficial process by suppressing adaptive signaling pathways [69]. Therefore, current evidence suggests that context, dosage, and temporal dynamics are critical determinants of biological outcome, and indiscriminate elimination of free radicals is not considered an optimal therapeutic strategy.

Although regulation of oxidative stress is fundamental for cellular survival, it does not resolve all aspects of cellular damage. Dysfunctional proteins, overloaded organelles, and impaired biosynthetic processes require more complex protective responses. When biochemical instability begins to affect protein quality control, the cellular crisis management center shifts toward the ER, where the UPR is activated. The mechanisms of UPR signaling will be discussed in the following subsection.

### 3.4. Endoplasmic Reticulum as the Cellular Crisis Center

If oxidative stress can be compared to chemical chaos, protein stress represents a crisis of production quality. The cell continuously synthesizes thousands of proteins that must undergo precise folding before acquiring functional conformations. ER serves as a specialized manufacturing site as well as a quality control center for this process. Any disturbance of homeostasis, including energy deficiency, oxidative stress, inflammation, or nutrient excess, can lead to the accumulation of misfolded proteins within the ER lumen.

UPR is not a single signaling pathway but an integrated adaptive program regulated by three membrane-anchored sensor proteins: PERK, IRE1, and ATF6 [70,71]. Under physiological conditions, these sensors are maintained in an inactive state through binding to the molecular chaperone BiP (GRP78).

When misfolded proteins accumulate, they sequester BiP away from the sensors, thereby triggering the stress response. PERK reduces the rate of new protein synthesis by phosphorylating the translation initiation factor eIF2α, allowing the ER to reduce folding load and restore proteostasis. IRE1 activates transcriptional programs that enhance protein folding capacity and promote degradation of damaged proteins while also participating in cross-talk with other cellular stress pathways. After translocation to the Golgi apparatus and proteolytic activation, ATF6 functions as a transcription factor that increases chaperone production, thereby strengthening the protein folding machinery.

A key feature of UPR is the transient suppression of protein synthesis. Although this may appear disadvantageous at first glance, it represents a highly rational adaptive strategy. Instead of continuing the production of potentially defective molecules, the cell reallocates resources toward repairing existing proteins and eliminating those that cannot be restored.

UPR signaling enhances the expression of molecular chaperones that assist proteins in achieving correct tertiary and quaternary structures. Simultaneously, it activates ERAD system, which directs irreversibly damaged proteins toward proteasomal degradation [72]. Through these mechanisms, ER functions as an intelligent production facility capable of dynamically adjusting operational speed and selectively removing defective products.

In this framework, the ER can be viewed as a cellular crisis command center that integrates signals from multiple intracellular pathways and determines whether adaptive restoration is feasible or whether activation of elimination mechanisms is necessary to preserve overall cellular integrity.

UPR functions as an adaptive mechanism only when stress exposure is transient. When ER overload persists for an extended period, the protective program gradually shifts toward a pro-apoptotic state. This transition reflects an evolutionarily conserved decision-making process in which cellular survival is weighed against the maintenance of tissue-level integrity.

A particularly important mediator of this transition is the transcription factor CHOP (C/EBP homologous protein), which promotes apoptosis by disrupting redox balance and regulating the expression of genes involved in programmed cell death [73]. CHOP activation contributes to mitochondrial dysfunction, oxidative stress amplification, and suppression of anti-apoptotic signaling pathways.

The shift from adaptive restoration to cellular elimination is fundamental for understanding the pathogenesis of many chronic non-communicable diseases. Chronic UPR activation can lead to the progressive loss of highly specialized, low-regenerative cell populations, such as neurons and pancreatic β-cells. Because these cells possess limited proliferative capacity, sustained ER stress may result in irreversible functional decline of tissues and organs, contributing to the development of neurodegenerative and metabolic disorders.

Growing evidence indicates that chronic ER stress and UPR dysregulation play significant roles in the development of chronic non-communicable diseases, including insulin resistance [74], type 2 diabetes [75], atherosclerosis [76], neurodegenerative disorders [77,78,79], liver diseases, and cancer progression [80].

The common denominator across these pathologies is persistent overload of the protein quality control system. Such overload may arise from excessive nutrient availability, chronic inflammation, or accumulation of pathogenic protein aggregates. This perspective shifts the conceptualization of disease from an acute structural injury model toward a framework in which pathology is viewed as a long-term failure of adaptive regulatory mechanisms that were originally evolved to protect cellular integrity.

Consequently, therapeutic strategies targeting UPR modulation represent a promising research direction [81]. Contemporary studies emphasize that complete inhibition of UPR signaling is undesirable. Instead, the objective is to restore adaptive balance by shifting UPR activity toward protective signaling rather than pro-apoptotic outcomes.

Recent investigations [21] also highlight the interconnection between ER stress, autophagy, and immune regulation. Both pharmacological agents targeting ER sensor activity and naturally derived compounds that attenuate proteostasis overload are being explored. Such approaches are studied particularly in the context of type 2 diabetes, neurodegenerative diseases, and chronic inflammatory disorders, where restoration of cellular adaptive capacity may offer therapeutic benefit.

### 3.5. Mitochondria—Powerhouses, Sensors, and Guardians of Cellular Quality

Mitochondria are not static, isolated organelles but form a highly dynamic network capable of continuous remodeling. One of the principal adaptive mechanisms is the balance between mitochondrial fusion and fission [82].

Fusion enables the redistribution of mitochondrial components, including proteins and mitochondrial DNA, thereby diluting localized molecular damage. Through content mixing, functionally impaired regions can be compensated by healthier mitochondrial segments. In contrast, fission facilitates the segregation of damaged mitochondrial fragments, allowing their selective removal via mitophagy.

This dynamic remodeling allows the cell to maintain energy production while simultaneously identifying and eliminating dysfunctional structures. The equilibrium between fusion and fission processes is therefore essential for mitochondrial quality control. Disruption of this balance promotes the accumulation of defective mitochondria and is associated with neurodegenerative disorders, aging processes, and insulin resistance [27].

When repair is no longer feasible, cells activate mitophagy, a specialized form of autophagy dedicated to the removal of irreversibly damaged mitochondria [83]. This process represents a high-cost adaptive strategy: the cell deliberately sacrifices a portion of its energetic capacity to prevent more severe systemic damage.

Mitophagy is tightly regulated by molecular tagging systems that mark dysfunctional mitochondria for degradation. One of the best-characterized pathways involves the PINK1-Parkin signaling axis, which accumulates on depolarized mitochondrial membranes and promotes the formation of autophagosomes around damaged organelles.

Functionally, mitophagy serves several protective roles. It limits excessive ROS production, preserves metabolic efficiency, and prevents activation of apoptotic cascades. Impairment of mitophagy leads to the accumulation of dysfunctional mitochondria. Such dysfunction is particularly detrimental to highly energy-dependent cells, including neurons and cardiomyocytes, where sustained mitochondrial quality control is essential for survival and proper physiological function.

Mitochondria also function as biochemical regulators that determine cellular fate under extreme stress conditions. Prolonged metabolic, oxidative, or proteotoxic overload may activate programmed cell death pathways. Although this process contributes to cell loss, it may limit propagation of irreparable cellular damage.

This regulatory process is primarily mediated through mitochondrial control of apoptosis. When stress signaling exceeds the adaptive capacity of the cell, mitochondrial outer membrane permeabilization is induced, resulting in the release of pro-apoptotic factors, including cytochrome c. Subsequent activation of caspase-dependent signaling cascades commits the cell to controlled self-destruction, thereby limiting inflammatory injury and preserving overall tissue homeostasis.

Chronic stress leads to gradual exhaustion of cellular quality control systems, impairment of mitophagy, and accumulation of dysfunctional mitochondria. As a consequence, mechanisms that initially served a protective function may begin to contribute to cellular dysfunction. The transition from adaptive responses to functional failure represents one of the key turning points in the pathogenesis of chronic non-communicable diseases. Mitochondrial dysfunction is among the most well-documented pathogenic factors in chronic diseases [12].

In type 2 diabetes, mitochondrial metabolic flexibility is reduced, accompanied by excessive ROS production. In atherosclerosis, damaged mitochondria within endothelial cells and macrophages amplify inflammatory signaling. In neurodegenerative disorders, disturbances in mitophagy and mitochondrial dynamics often precede the onset of clinical symptoms.

Increasing evidence suggests that mitochondria are not merely passive targets of disease processes but active contributors to pathogenesis. This has stimulated interest in mitochondria-targeted antioxidants designed to neutralize ROS at their site of generation while preserving physiological redox signaling.

In parallel, strategies aimed at supporting mitochondrial quality control are being developed, including interventions that improve mitochondrial dynamics and modulate mitophagy. The primary therapeutic objective is not maximal ATP production but the maintenance of a functionally competent and metabolically adaptable mitochondrial population.

Mitochondria therefore illustrate that the cellular response to stress does not rely on its complete avoidance. Cellular health is based on the capacity for flexible adaptation, the efficient management of biological costs, and the ability to recognize when continued functioning becomes unsafe. This represents a delicate equilibrium between survival and the preservation of organismal integrity.

Mitochondria demonstrate that cellular health is not achieved by eliminating stress but by maintaining adaptive responsiveness to it. Physical activity [84,85], periodic caloric restriction [86], and even moderate metabolic stress can stimulate mitochondrial biogenesis and enhance mitochondrial quality control mechanisms.

This apparent paradox, that controlled stress can strengthen cellular resilience, will be a central theme in the subsequent section of this work, which focuses on lifestyle-related modulation of biological stress responses.

### 3.6. Autophagy—Cellular Cleaning, Repair, and Homeostatic Restoration

Cellular survival requires not only the synthesis of new molecules but also the continuous removal of damaged, dysfunctional, or unnecessary components. Proteins may lose their structural integrity, organelles may become impaired, and molecular aggregates may interfere with system-level cellular function. Autophagy represents the cellular response to this challenge, a universal “cleaning” mechanism that simultaneously serves adaptive and protective roles.

Although the term autophagy literally means “self-eating”, the process is not primarily destructive. Instead, it is a precisely regulated recycling system that allows the cell to recover amino acids, fatty acids, and nucleotides from degraded cellular components [87].

In classical macroautophagy, a cytoplasmic region containing damaged proteins or organelles is enveloped by a double-membrane structure, forming an autophagosome. This vesicle subsequently fuses with a lysosome, whose hydrolytic enzymes break down the enclosed material into simple metabolites that can be reused. The process is tightly controlled by autophagy-related genes (ATG) and signaling pathways that respond to cellular energetic status. A key regulatory component is the mTOR kinase, which functions as a cellular “well-being sensor”. When nutrient availability is high, autophagic activity is suppressed. Under conditions of nutrient deprivation, oxidative stress, or ER overload, autophagy is activated as a rescue mechanism.

Autophagy plays a central role in maintaining proteostasis by systematically eliminating cytotoxic aggregates and damaged cellular structures, thereby preventing the initiation of chronic inflammatory processes at the cellular level [88].

Autophagy does not operate in isolation but is functionally interconnected with other stress response systems described previously. ER stress and activation of the UPR increase the demand for removal of misfolded proteins, thereby stimulating autophagic activity. Dysfunctional mitochondria are selectively eliminated through mitophagy, a specialized form of mitochondrial autophagy. Excess ROS can also induce autophagy as a protective response against oxidative damage. Consequently, autophagy represents a convergent adaptive mechanism integrating metabolic, redox, and proteostatic signals across multiple cellular stress pathways.

Growing evidence indicates that impaired autophagy contributes to the pathogenesis of many chronic diseases. In obesity and type 2 diabetes, reduced autophagic activity is observed in metabolically active tissues, promoting the accumulation of dysfunctional mitochondria and exacerbating insulin resistance [89,90].

In atherosclerosis, impaired autophagy in macrophages contributes to the formation of foam cells and destabilization of atherosclerotic plaques. The role of autophagy is particularly prominent in neurodegenerative disorders [91,92]. Neurons, being long-lived and largely non-proliferative cells, are especially dependent on efficient cellular clearance systems. The accumulation of protein aggregates in conditions such as Alzheimer’s or Parkinson’s disease is largely associated with dysfunction of autophagic and lysosomal pathways.

Pharmacological attempts to directly stimulate autophagy are ongoing, although such interventions require considerable caution. In clinical practice, greater promise currently lies in indirect modulation of autophagic activity, for example, through regulation of energy metabolism, circadian rhythm, or hormonal signaling pathways. This approach aims to restore physiological autophagic dynamics rather than forcing excessive activation of degradation processes.

Although autophagy is generally considered a protective mechanism, excessive or dysregulated autophagic activity may become detrimental. In certain cancers, autophagy can support the survival of malignant cells under hypoxic conditions and nutrient deprivation [93]. For this reason, autophagy is currently viewed not as inherently beneficial or harmful, but as a process requiring precise regulatory balance. This perspective reflects a fundamental principle of stress biology: cellular fate is determined not by the presence of stress itself, but by the capacity to regulate and integrate stress responses.

The biological significance of autophagy gained widespread recognition in 2016, when the Nobel Prize in Physiology or Medicine was awarded to Yoshinori Ohsumi for elucidating the molecular mechanisms underlying this process [88]. Initially studied in yeast models, autophagy was shown to be evolutionarily conserved across species, from unicellular organisms to humans. Today, autophagy represents one of the most intensively investigated biological processes, linking aging, metabolism, immunity, and chronic disease into a unified conceptual framework.

### 3.7. Genome Repair Systems—Protecting the Cellular Instruction Manual

DNA can be compared to the central instruction manual of the cell. It contains highly precise information governing protein synthesis, metabolic regulation, and cellular identity. However, unlike a secure archive, DNA is continuously exposed to damage arising from normal metabolic activity, ROS, ultraviolet radiation, and replication errors [1].

Damage to DNA represents not only a structural defect but also a danger signal for the cell. Single- or double-strand breaks, nucleotide modifications, and replication mismatches are rapidly detected by sensor proteins. These molecular detectors function to halt cell cycle progression, allowing time for repair processes to operate.

A central role in this response is played by the ATM and ATR kinases, which act as molecular crisis coordinators [94]. Upon activation, they initiate signaling cascades that suppress cell division, stimulate DNA repair enzymes, and, in cases of severe or irreparable damage, trigger programmed cell death (apoptosis). This hierarchical response preserves genomic stability and prevents the propagation of potentially malignant mutations.

DNA damage response (DDR) does not always lead to successful repair. If genomic lesions are too extensive or recurrent, the cell may enter a state of permanent cell cycle arrest known as senescence or initiate apoptosis. This represents a protective mechanism at the level of the organism, preventing the proliferation of cells carrying unstable or potentially oncogenic genomes.

Problems arise when DDR signaling becomes inefficient or chronically activated. Impaired repair processes promote mutation accumulation, whereas sustained DDR activation contributes to cellular aging and the secretion of pro-inflammatory factors. In both scenarios, tissue function gradually deteriorates.

ROS generated in mitochondria are among the major endogenous sources of DNA damage. Conversely, damaged DNA, particularly when genetic fragments are released into the cytoplasm, may activate immune signaling pathways. In this way, oxidative stress, mitochondrial dysfunction, and DDR form a self-reinforcing feedback loop that promotes chronic inflammation. This mechanism is increasingly recognized as a fundamental component in the pathogenesis of chronic non-communicable diseases.

One of the most consistent findings in aging biology is the progressive decline in genome repair efficiency [95]. With advancing age, the expression of key DNA repair enzymes decreases, oxidative stress levels increase, and cells more frequently shift toward senescence rather than efficient regeneration. The consequence is growing genomic instability, a hallmark shared by aging, cancer, neurodegenerative disorders, and metabolic diseases. Many age-related pathologies can therefore be interpreted as manifestations of an accumulating “genetic debt”.

Modern medicine increasingly utilizes knowledge of DDR mechanisms in clinical practice. In oncology, targeted inhibition of selected DNA repair pathways enhances the sensitivity of cancer cells to chemotherapy and radiotherapy [96]. Conversely, in the context of aging and chronic disease, strategies aimed at supporting physiological genome repair are being actively explored [97]. DDR is thus no longer viewed solely as a molecular repair system but as a central component of the biological narrative explaining how cells cope with long-term stress and why this adaptive struggle may sometimes fail.

## 4. When the System Fails: Cellular Stress as the Basis of Chronic Non-Communicable Diseases

A key determinant in the pathogenesis of chronic non-communicable diseases is the duration of stress exposure. Chronic overload leads to a transition from adaptive responses to maladaptive states [98]. In this condition, cellular defense systems become progressively overwhelmed, resulting in loss of homeostasis, accumulation of molecular damage, and disease progression.

At the organismal level, this state is characterized by persistent low-grade inflammation, endocrine dysregulation, and gradual organ dysfunction. Cells progressively lose regenerative capacity, while degenerative processes begin to dominate over repair mechanisms. Increasing evidence suggests that many chronic non-communicable diseases can be interpreted as different phenotypic manifestations of a single fundamental biological problem: chronic cellular stress [99]. The phenotypic heterogeneity of these disorders arises from the dominant type of stressor, tissue-specific vulnerability, and genetic background.

Prolonged stress also triggers the release of stress hormones, including cortisol, adrenaline, and noradrenaline. These hormones increase metabolic rate, elevate oxygen consumption within mitochondria, and accelerate cellular respiration. As metabolic activity intensifies, the generation of ROS increases as a natural by-product of oxidative phosphorylation, further contributing to oxidative stress and molecular damage. This creates a feedback relationship in which stress signaling and metabolic acceleration reinforce one another.

### 4.1. ER Stress in Pancreatic β-Cells

Oxidative stress, a major pathogenic factor in diseases such as diabetes, obesity, and diabetes-associated microvascular complications, arises from excessive intracellular ROS accumulation [100]. Diabetes is characterized by dysfunction or loss of pancreatic β-cells responsible for insulin secretion, resulting either from autoimmune destruction (type 1 diabetes) or from inadequate compensation for insulin resistance (type 2 diabetes).

ER serves as the primary site of proinsulin folding; therefore, ER proteostasis is essential for both the function and survival of β-cells under physiological and pathological conditions. The ER also functions as a major intracellular calcium storage compartment, generating Ca^2+^-dependent signals that regulate insulin secretion.

ER stress is closely associated with diabetes pathogenesis. To maintain sufficient insulin granule reserves in the context of continuous metabolic demand, β-cells must synthesize large amounts of proinsulin, the precursor of insulin. Proinsulin biosynthesis may account for approximately 30–50% of total protein synthesis in β-cells. This high synthetic burden places significant pressure on the secretory pathway, particularly the ER, where proinsulin undergoes initial folding, including the formation of three evolutionarily conserved disulfide bonds. In normal β-cells, up to approximately 20% of newly synthesized proinsulin may fail to achieve its native conformation, indicating that proinsulin is intrinsically prone to misfolding.

Misfolded proinsulin molecules may either be refolded into their native structure or degraded through ERAD pathways and autophagy. While such degradation reduces proinsulin processing efficiency, it does not otherwise impair β-cell function under physiological conditions. However, under certain pathological states, the rate of proinsulin misfolding increases beyond the genetically determined threshold that β-cells can compensate for. This leads to progressive accumulation of misfolded proinsulin within the ER, a phenomenon considered a causal factor in β-cell failure and the development of diabetes [101].

### 4.2. Lipotoxicity and Glucotoxicity as Sources of Metabolic Stress

Metabolic stress can be defined as a condition in which cells are exposed to energy substrates that are either excessive or insufficient relative to their metabolic demands. The primary nutrients that may exert toxic effects on cells through ER dysfunction are free fatty acids (FFAs) and glucose.

Oxidation of FFAs released from triglycerides derived from dietary fats represents the principal mechanism by which peripheral tissues meet their energy requirements. Under conditions of excess fatty acid availability, FFAs are stored in adipocytes for later use during periods of fasting. However, circulating FFA levels increase when fatty acid supply exceeds the storage capacity of adipose tissue. Such excess may result in lipid accumulation within cells and tissues that lack appropriate mechanisms for lipid processing and storage, ultimately leading to cellular damage.

Recent research suggests that the toxic effects of saturated fatty acids, such as palmitate (C16:0), may be associated with their excessive incorporation into cellular membranes, including the ER membrane. This process can reduce the relative abundance of essential lipids such as sphingomyelin and cholesterol, which are required for optimal ER function. Consequently, disruption of membrane lipid composition contributes to lipotoxicity in pancreatic β-cells [102]. Furthermore, elevated plasma cholesterol levels may induce ER lipotoxicity by altering membrane structure and stability [103]. The ER is highly sensitive to cholesterol concentration and plays a key role in regulating intracellular cholesterol metabolism.

### 4.3. The Role of Oxidative Stress in LDL Oxidation

Lipoprotein oxidation is a critical early event in the development of atherosclerosis, a disease characterized by the formation of atherosclerotic plaques within arterial walls. Oxidation of low-density lipoprotein (LDL) has been demonstrated to be a key factor in atherogenesis.

Oxidized LDL (Ox-LDL) exhibits several atherogenic properties, contributing to endothelial dysfunction, foam cell formation, and inflammatory activation within the arterial wall. Interaction between Ox-LDL and specific endothelial cell receptors plays a central role in these pathological processes [104]. Increased ROS production promotes the oxidative modification of LDL components, including phospholipids, cholesterol esters, and polyunsaturated fatty acids, ultimately leading to Ox-LDL formation.

LDL oxidation is a complex, multistage process. The initiation phase involves free radical attack on polyunsaturated fatty acids present within LDL particles, generating lipid peroxyl radicals. This is followed by the propagation phase, during which these radicals react with additional fatty acids, establishing a chain reaction that results in widespread lipid peroxidation. The process concludes with the termination phase, in which antioxidants or other reactive molecules neutralize lipid radicals and halt oxidative progression [105,106]. In scenarios characterized by insufficient antioxidant defense, Ox-LDL particles are recognized and internalized by macrophages, leading to foam cell formation, a hallmark feature of atherosclerotic lesion development.

### 4.4. Inflammation as a Driver of Cellular Stress in the Vascular Wall

Chronic inflammation, which often begins as a biological response to vascular endothelial dysfunction, is considered a primary driver of atherosclerosis. Factors such as oxidative stress, Ox-LDL, thrombosis, and viral or bacterial infections induce both acute and chronic inflammatory cell infiltration, including neutrophils, lymphocytes, and macrophages. These infiltrating immune cells further amplify local vascular inflammation through increased cytokine production [107,108,109]. The coexistence of cardiovascular disease and chronic inflammation establishes a self-perpetuating feedback loop that contributes to the persistence and progression of atherosclerotic lesions.

ROS are strongly implicated in vascular inflammation. Excessive ROS production activates intracellular signaling pathways, including redox-sensitive transcriptional regulators, resulting in increased expression of adhesion molecules, elevated chemokine concentrations, enhanced cytokine synthesis, and monocyte infiltration into the vascular wall [110,111]. Moreover, inflammatory activation enhances inducible nitric oxide synthase expression, increasing RNS generation.

Numerous studies have demonstrated that inflammatory processes contribute to vascular injury [112,113,114,115]. The presence of angiotensin II, Ox-LDL, and pro-inflammatory cytokines activates NADPH oxidase, which further promotes oxidative stress-driven inflammation through excessive ROS generation.

Consequently, oxidative stress, inflammation, and endothelial dysfunction are closely interconnected and play central roles in the initiation, progression, and maintenance of atherosclerosis. These processes mutually reinforce one another, forming a pathological cascade in which ROS induce cellular injury and trigger inflammatory signaling, while cytokines and chemokines recruit immune cells, including macrophages and neutrophils, to sites of inflammation, leading to additional ROS and cytokine release. In the central nervous system, microglia and astrocytes play key roles in inflammatory responses and ROS production [116].

### 4.5. Neurodegeneration

The nervous system is highly susceptible to oxidative stress. Both peripheral nerves and the central nervous system (CNS) are rich in lipids and mitochondria, which are particularly vulnerable targets of oxidative damage [117]. The brain is the most oxygen-demanding organ in the body, consuming approximately 20% of total oxygen supply, which contributes to increased ROS generation [118,119]. The high energetic demand of the brain is largely dependent on mitochondrial function, and enhanced mitochondrial activity is associated with greater ROS production as a metabolic by-product. Additionally, the abundance of unsaturated fatty acids in the CNS facilitates oxidative attack by free radicals.

Neurons are generally non-dividing cells, meaning that oxidative damage to neurons is largely irreversible. Beyond intrinsic vulnerability to oxidative stress, the brain also possesses relatively limited antioxidant capacity. For example, neuronal catalase and GSH activity are significantly lower than in other tissues; neuronal catalase levels have been reported to be approximately 50-fold lower than those observed in hepatocytes [120]. This reduced antioxidant defense further lowers the threshold for oxidative injury in neural tissue.

### 4.6. Protein Aggregates, ER Overload, and Autophagy Dysfunction

Protein aggregation is a common hallmark of disorders associated with protein misfolding, also referred to as conformational diseases, including the majority of neurodegenerative conditions. These disorders represent a major and growing clinical burden for which effective disease-modifying therapies remain largely unavailable. Increasing evidence indicates that ER stress constitutes a principal mechanism of cytotoxicity in these diseases. Whether involving β-amyloid or tau protein in Alzheimer’s disease, α-synuclein in Parkinson’s disease, huntingtin in Huntington’s disease, or other aggregation-prone proteins implicated in various neurodegenerative disorders, a shared molecular pathway can be identified. This pathway involves progressive oligomerization and aggregation into amyloid fibrils [121].

Alzheimer’s disease (AD) is the most common neurodegenerative disorder among older individuals and is characterized by progressive cognitive decline with memory impairment. Two principal pathological hallmarks define AD: extracellular plaques composed of aggregated β-amyloid (Aβ) peptides and intracellular neurofibrillary tangles formed by hyperphosphorylated microtubule-associated tau protein. Both pathological features are closely associated with the induction of oxidative stress [116].

Similarly, the accumulation and aggregation of α-synuclein in Lewy bodies and Lewy neurites within the central nervous system, forming amyloid fibrils, play a central role in the pathophysiology of Parkinson’s disease (PD) and in a subgroup of neurodegenerative disorders collectively referred to as Lewy body dementias [122].

Primary lysosomal storage resulting from inherited lysosomal defects may facilitate the initial accumulation of amyloidogenic proteins within the lysosomal compartment. Such accumulation further compromises neuronal autophagic and lysosomal degradation capacity through multiple mechanisms, potentially including impaired trafficking of lysosomal enzymes. These processes establish a self-reinforcing pathological loop that accelerates neurodegenerative progression [123].

### 4.7. Mitochondrial Dysfunction in Neurons

Progressive impairment of mitochondrial function is widely regarded as a major contributor to increased ROS production. Mitochondrial performance declines gradually with aging and in AD, leading to reduced ATP synthesis and enhanced ROS generation. This, in turn, further compromises energy supply and exacerbates mitochondrial dysfunction, establishing a self-perpetuating cycle of bioenergetic failure [124].

ROS also impair mitochondrial biogenesis and disrupt mitochondrial dynamics, resulting in defects in mitochondrial fission and fusion processes [125]. Importantly, restoration of balanced mitochondrial dynamics has been shown to reduce mitochondrial superoxide levels [126]. Evidence presented by Leuner et al. [127] suggests that mitochondria-derived ROS contribute directly to mitochondrial dysfunction and to the pathogenesis of AD. During aging, antioxidant defense systems decline, while ROS production increases, leading to a progressive rise in oxidative stress. Mitochondrial dysfunction is considered a hallmark of aging. Age-related functional decline of mitochondria reduces cellular energy availability, promotes cellular damage, and further enhances ROS production [128].

### 4.8. Psychological and Cellular Stress

Psychological stress represents a complex organismal response to stimuli perceived as threatening to homeostasis. Although its primary origin is cognitive and emotional, its consequences involve multi-level physiological alterations, including activation of the hypothalamic–pituitary–adrenal (HPA) axis, stimulation of the sympathetic nervous system, and induction of inflammatory processes [129]. From a psychobiological perspective, psychological stress arises when a situation is perceived as exceeding an individual’s adaptive resources. In response, central regulatory systems are engaged to restore equilibrium. Chronic activation of these systems leads to what is termed allostatic load, a cumulative burden that produces long-term molecular and cellular changes [129].

A central mechanism of the stress response is activation of the HPA axis [130]. In response to stress, the hypothalamus secretes corticotropin-releasing hormone (CRH), which stimulates the anterior pituitary to release adrenocorticotropic hormone (ACTH). ACTH subsequently induces glucocorticoid secretion—primarily cortisol—from the adrenal cortex. Under physiological conditions, cortisol exerts multiple regulatory effects through complex mechanisms, including binding to intracellular glucocorticoid receptors (GR) and modulation of gene transcription [131]. Cortisol plays a central role in maintaining homeostasis and exerts anti-inflammatory actions, including regulation of leukocyte trafficking and suppression of pro-inflammatory cytokine secretion [132,133]. Additionally, cortisol potentiates catecholamine activity and further shapes immune responses through modulation of cytokine production [134].

Under chronic stress conditions, dysregulation of the HPA axis may occur, resulting in persistent hypercortisolemia or, paradoxically, secondary hypocortisolemia following prolonged overstimulation. Sustained exposure to stress mediators induces a range of pathophysiological adaptations, including reduced receptor sensitivity and stable epigenetic modifications such as DNA methylation. These changes create a long-lasting biochemical “imprint” of stress within cells [135], potentially disrupting nocturnal regenerative processes and accelerating biological aging [136]. Chronic HPA axis dysfunction and its downstream effects on immune function have been associated with increased susceptibility to infections, delayed wound healing, heightened risk of autoimmune diseases (e.g., rheumatoid arthritis, systemic lupus erythematosus), and elevated cardiovascular risk mediated by persistent low-grade inflammation [137,138]. One contributing mechanism may involve attenuation of the tolerogenic effects of glucocorticoids on dendritic cells during HPA axis dysfunction, reducing the immune system’s capacity to distinguish self from non-self [139,140]. As a consequence, the immune environment may shift from tolerance toward autoimmunity, clinically manifesting as the onset or exacerbation of autoimmune conditions such as multiple sclerosis, rheumatoid arthritis, or systemic lupus erythematosus [139,140].

## 5. Lifestyle as a Modulator of Cellular Stress

Lifestyle is often conceptualized as a collection of individual choices concerning diet, physical activity, and social functioning. From the perspective of molecular biology, however, it constitutes a continuous stream of biochemical signals that dynamically reshape metabolism, gene expression, and the adaptive limits of cells. Environmental inputs, ranging from nutrient density and circadian synchronization to subjective stress perception, are integrated by interconnected signaling networks that determine, in real time, whether homeostasis is preserved or whether the system shifts toward decompensation [141,142].

A central framework for understanding this relationship is hormesis. This nonlinear dose–response phenomenon demonstrates that low-intensity stress is not inherently destructive; rather, it serves as a critical stimulus that activates repair and protective mechanisms. Only excessive or sustained stress leads to defensive system overload, depletion of adaptive reserves, and progressive damage. At the cellular level, a dynamic equilibrium exists between anabolic/growth-promoting and regenerative/stress-responsive signaling pathways. Lifestyle factors actively shift this balance, modulating the threshold between adaptation and maladaptation [143].

Within this conceptual model, lifestyle ceases to be merely a physiological background or a matter of preference and instead emerges as a direct epigenetic modulator [144]. Daily behavioral patterns influence chromatin remodeling, DNA methylation, histone modifications, and non-coding RNA regulation, thereby shaping transcriptional responsiveness and stress resilience. Consequently, routine behaviors function as biologically active determinants that modulate cellular robustness against the cumulative stressors characteristic of contemporary chronic non-communicable diseases [145].

### 5.1. Diet

In the traditional view, food is primarily regarded as a source of energy and structural substrates. From the perspective of molecular biology, however, dietary components exert far broader functions: they serve as critical signaling molecules that directly influence mitochondrial activity and cellular metabolism. Within this framework, each meal provides specific biochemical information, initiating signaling cascades that determine whether the organism prioritizes growth and energy storage or shifts toward regeneration and cytoprotective processes [146].

Metabolism thus emerges as an integrated regulatory system in which distinct nutrients shape cellular integrity. This influence spans multiple levels of biological organization. The qualitative composition of fatty acids determines membrane fluidity, receptor dynamics, and mitochondrial membrane function. Glycemic excursions affect proteome stability and increase the risk of non-enzymatic glycation, thereby contributing to structural and functional protein impairment. Periodic energy deficits, in turn, activate enzymatic repair and stress-adaptation pathways, including AMP-activated protein kinase (AMPK) signaling, autophagy, and mitochondrial biogenesis.

Through these mechanisms, diet continuously remodels the intracellular environment, functioning as an active metabolic regulator. Depending on its qualitative and quantitative characteristics, nutritional input may reinforce physiological homeostasis or, under conditions of chronic caloric excess and nutrient imbalance, promote metabolic inflexibility, oxidative stress, and progressive cellular destabilization [147].

A central component of metabolic regulation is carbohydrate homeostasis, in which glucose serves as a dominant, yet potentially highly reactive, metabolic fuel. Under physiological conditions, excess glucose is preferentially converted and stored as triglycerides within adipose tissue, representing a protective mechanism against acute hyperglycemia. However, chronic energy surplus leads to progressive adipocyte hypertrophy and eventual exhaustion of safe storage capacity, creating a pro-inflammatory milieu that promotes systemic insulin resistance [148,149].

Adequate dietary protein intake may exert a buffering effect in this context. Amino acids not only support the maintenance and expansion of lean body mass but also modulate postprandial glucose kinetics by slowing gastric emptying and attenuating rapid glucose absorption. This contributes to stabilization of insulin secretion dynamics and reduces acute glycemic excursions, thereby limiting metabolic stress at the cellular level [150].

When compensatory mechanisms fail, metabolic homeostasis deteriorates, resulting in dysregulated intracellular substrate flux. Persistent hyperglycemia promotes non-specific and pathological interactions between glucose and structural biomolecules, including proteins and lipids [151]. A critical process in this setting is glycation—the non-enzymatic condensation of reducing sugars with free amino groups in proteins. This reaction leads to the formation of Advanced Glycation End-products (AGEs), stable and often biologically dysfunctional molecular aggregates [152].

Accumulation of AGEs compromises vascular elasticity, impairs enzymatic activity, and disrupts the structural integrity of tissues. Cross-linking of extracellular matrix proteins, altered receptor signaling via RAGE (receptor for AGEs), and amplification of oxidative stress further propagate inflammatory signaling cascades. Consequently, uncontrolled carbohydrate influx ceases to be merely an energetic imbalance and becomes a mechanism of progressive structural damage, forming a molecular basis for chronic low-grade inflammation and metabolic disease development [153].

In contrast to chronic energy surplus, periodic caloric restriction and short-term fasting constitute potent stimuli that activate evolutionarily conserved survival programs. Although both approaches are based on negative energy balance, complete and time-limited food withdrawal appears particularly effective in optimizing insulin dynamics, thereby enabling sustained suppression of the mTOR pathway, a central regulator of anabolic processes and cell proliferation [154,155].

Under these conditions, metabolic priorities shift from tissue expansion toward preservation and optimization of existing cellular structures. Increased activation of AMPK induces systemic metabolic reprogramming, redirecting energy utilization from biosynthetic pathways toward repair processes, autophagy, and enhanced resistance to oxidative stress [156]. In this framework, energy restriction is not merely a reduction in substrate availability but a regulatory signal that compels cells to reassess structural integrity and functional efficiency, thereby reinforcing stress resilience mechanisms.

An important complement to caloric restriction strategies is the action of polyphenols, which function as mild stress mimetics. Their biological effects extend beyond direct free radical scavenging. Instead, polyphenols act as hormetic stimuli that activate endogenous cytoprotective pathways, including nuclear factor erythroid 2-related factor 2 (Nrf2), sirtuins, and mitochondrial biogenesis signaling cascades [157,158]. Through these mechanisms, polyphenols do not simply provide exogenous antioxidant protection; rather, they systemically modulate adaptive responses, priming cells for improved management of oxidative stress, mitochondrial dysfunction, and age-associated molecular damage.

### 5.2. Sleep—Architecture of Nocturnal Cellular Repair

Modern biochemistry does not regard sleep as a passive state of rest but as a phase of highly coordinated molecular homeostasis. Sleep represents a chronobiologically regulated process during which cellular metabolic priorities are fundamentally reorganized [159]. Energy resources that are primarily allocated during daytime to anabolic activity and environmental interaction are redirected toward endogenous repair mechanisms, genomic stability surveillance, and the removal of neurotoxic metabolic by-products [159,160].

The cornerstone of nocturnal regeneration is the circadian rhythm system, governed by a hierarchical network of molecular oscillators. A central regulatory role is played by the CLOCK-BMAL1 protein complex, which functions as a master transcriptional regulator of clock-controlled genes [161]. This system is synchronized with environmental light–dark cycles through signals such as the nocturnal release of melatonin, which may also support endogenous antioxidant capacity [162]. Through this integrated regulatory network, the temporal expression of thousands of genes involved in glucose metabolism and DNA repair is precisely coordinated. Disruption of this synchronization, such as exposure to short-wavelength blue light during nighttime, may induce molecular desynchronization, suppressing critical regenerative programs despite apparent physical rest of the organism [161,163]. Protein folding is highly sensitive to energy availability and temporal stability; therefore, sleep deprivation can induce ER stress. During deep restorative sleep, specialized molecular chaperones are activated to monitor and maintain the correct three-dimensional conformation of newly synthesized polypeptide chains. If protein folding proceeds improperly, the UPR is triggered as an adaptive quality-control mechanism [18,164]. Under conditions of adequate sleep, this pathway contributes to the restoration of proteostasis by repairing misfolded proteins or directing irreversibly damaged molecules toward degradation. However, chronic impairment of nocturnal regeneration overloads cellular repair systems, promoting the accumulation of toxic protein aggregates. The removal of such aggregates becomes a priority for neural clearance mechanisms, particularly those associated with glial-mediated tissue maintenance in the central nervous system [165].

Clearance of neurotoxic metabolic by-products from the central nervous system occurs through the glymphatic system, a specialized perivascular pathway whose activity is tightly regulated by the sleep–wake cycle [166]. This mechanism relies on convective cerebrospinal fluid flow through brain parenchyma, facilitated by aquaporin-4 (AQP4) water channels located in astrocytic end-feet [166]. The efficiency of glymphatic drainage reaches its peak during slow-wave sleep (non-rapid eye movement, NREM sleep), when reduced noradrenergic signaling leads to a substantial expansion of the extracellular space [167]. This structural and neurochemical state significantly lowers tissue resistance, enabling more effective removal of protein aggregates, including β-amyloid and tau proteins, thereby protecting neurons from proteotoxic damage. Sleep deprivation constitutes a direct mechanical and physiological barrier to glymphatic function. Insufficient sleep promotes retention of neurotoxic metabolites and contributes to progressive deterioration of the neuronal microenvironment [168]. Sleep represents a critical temporal window for maintaining genomic integrity in neurons [160]. During wakefulness, high metabolic activity and increased ROS production contribute to the accumulation of molecular damage, including strand breaks and other structural disruptions of DNA continuity [169]. During the resting phase, the activity of DNA repair enzymes increases markedly. Among these, poly(ADP-ribose) polymerase 1 (PARP1) plays a central role. PARP1 functions as a molecular damage sensor that detects DNA helix destabilization and initiates the recruitment and assembly of repair complexes at sites of genomic injury. The activity of PARP1 is tightly coupled to circadian regulation and requires nicotinamide adenine dinucleotide (NAD^+^) as a critical substrate. Consequently, the efficiency of nocturnal DNA repair is directly influenced by cellular metabolic status [170].

Effective functioning of these mechanisms prevents the fixation of mutations and chromosomal abnormalities, which are considered major biomarkers of biological aging [160]. Chronic sleep deprivation leads to collapse of the protective mechanisms described above, resulting in rapid destabilization of cellular homeostasis. The inability to conduct nocturnal repair programs contributes not only to progressive DNA fragmentation but also to the induction of sterile inflammatory states [171]. This process occurs in the absence of pathogenic infection. Instead, it is triggered by the release of damaged DNA fragments and structurally altered proteins into the cytosol, where they are recognized by the immune system as danger signals, known as damage-associated molecular patterns (DAMPs) [171]. As a consequence, persistent activation of pro-inflammatory signaling pathways occurs, accelerating tissue degeneration and contributing to impaired cognitive function.

### 5.3. Physical Activity—A Hormetic Growth Stimulus

Traditional perspectives on physical activity primarily emphasize energy expenditure; however, from a biochemical standpoint, exercise represents a form of eustress—a beneficial stress stimulus. In contrast to chronic oxidative stress associated with hyperglycemia, the transient increase in ROS during exercise does not exert destructive effects. Instead, it functions as a precise signaling mechanism that mobilizes cellular resources and shifts cells into a state of enhanced adaptive readiness [172].

During muscle contraction, ROS production increases substantially. This phenomenon, known as the oxidative stress paradox, serves as a necessary trigger for activating endogenous cellular defense systems [173]. Short-term exposure to ROS stimulates specific signaling pathways that strengthen intrinsic antioxidant capacity more effectively than exogenous antioxidant supplementation with vitamins [174].

This adaptive response enhances cellular resilience not only to post-exercise metabolic stress but also to a broader spectrum of environmental stressors, thereby contributing to the development of systemic biological reserve capacity [175]. A key element of exercise adaptation is the remodeling and expansion of the mitochondrial network. Through this process, skeletal muscle cells not only improve their energy production efficiency but also enhance overall metabolic flexibility [176]. The formation of a more extensive and densely interconnected mitochondrial network enables more effective regulation of energetic substrates. In the long term, this reduces the risk of uncontrolled electron leakage during oxidative phosphorylation and limits structural damage within organelles [177].

Contemporary exercise biochemistry indicates that skeletal muscle functions as a major interorgan communication system, exhibiting significant secretory activity [178]. During mechanical contraction, myocytes synthesize and release myokines into the circulation-signaling molecules with broad systemic regulatory effects [179]. Of particular importance in the context of aging processes is irisin, a myokine capable of crossing the blood–brain barrier. Irisin promotes the expression of brain-derived neurotrophic factor (BDNF), directly stimulating neuroplasticity and strengthening neuroprotective mechanisms [180]. This molecular signaling cascade has direct implications for mental function. Increased BDNF availability, together with modulation of neurotransmitter systems such as dopamine and serotonin, contributes to mood stabilization and maintenance of cognitive performance [181]. Through these mechanisms, physical activity extends beyond peripheral metabolic conditioning and emerges as a central regulator of central nervous system function and emotional homeostasis [182].

### 5.4. Psychological Stress—Molecular Trace of Emotion

Psychological stress extends beyond subjective perception and functions as a powerful driver of cellular biochemical remodeling. This phenomenon is mediated through neuroendocrine transduction, whereby cognitive and emotional stimuli are converted into specific intracellular signaling pathways capable of modifying metabolism and genomic stability [183].

From a cellular perspective, stress is not merely a behavioral response but is associated with systemic stress signaling and reallocation of cellular energy resources. Under chronic stress conditions, priority is often shifted away from long-term repair and regenerative processes toward immediate survival responses, which may accelerate cumulative molecular damage [184].

In contrast to the HPA axis, which initiates the acute stress response, the endocannabinoid system (ECS) functions as a higher-order autoregulatory network aimed at terminating stress signaling [185]. The ECS operates through retrograde neurotransmission. Endogenous lipid ligands, such as anandamide, are synthesized on demand in response to neuronal activation and subsequently diffuse backward across synapses to activate cannabinoid receptors CB1 and CB2 [186]. This mechanism functions as a biochemical safety circuit that selectively suppresses excitatory neurotransmitter release and contributes to stabilization of HPA axis activity [187].

However, chronic stress exposure can disrupt ECS regulation through enzymatic dysregulation, leading to accelerated degradation of endocannabinoids and depletion of adaptive signaling capacity [188]. Impairment of this system reduces the ability of cells and neural networks to return to homeostatic equilibrium, thereby promoting cellular aging processes and maintaining a pro-inflammatory metabolic phenotype [189]. The link between psychological burden and systemic immune response is mediated by direct bidirectional communication between the nervous system and immune cells [190]. Under chronic stress conditions, excessive neurotransmitter release, particularly norepinephrine, and persistent dysregulation of the HPA axis act as strong modulators of macrophage and monocyte activity. This state promotes intracellular assembly of multiprotein complexes known as inflammasomes, which stimulate the production and secretion of pro-inflammatory cytokines such as interleukin-6 (IL-6) and tumor necrosis factor-alpha (TNF-α). The resulting process induces sterile peripheral inflammation, reflecting the biochemical interpretation of stress as a tissue damage-associated signal [191]. Long-term persistence of these inflammatory mediators in the circulation contributes to inflammaging, a chronic low-grade inflammatory state that progressively compromises biological barrier function and accelerates systemic cellular degeneration [192]. This mechanism is considered a direct catalyst of accelerated biological aging [193].

The most measurable evidence of the impact of stress on physiological longevity is telomere shortening. Telomeres are protective nucleotide sequences located at chromosome ends and function as a molecular mitotic clock that preserves genomic integrity during DNA replication [194]. Chronic exposure to cortisol, together with associated oxidative stress, significantly reduces telomerase activity, the enzyme responsible for telomere maintenance and elongation [195]. As a consequence, cells chronically exposed to stress-related endocrine and oxidative signals exhibit accelerated replicative aging, reaching the Hayflick limit earlier and entering a state of cellular senescence. This subcellular process contributes to reduced biological resilience and premature decline in tissue regenerative capacity relative to chronological age [195].

### 5.5. Impact of Lifestyle on Cellular Stress

Analysis of the molecular mechanisms described in this section suggests that aging is not merely a linear consequence of time passage but rather a result of continuous adaptive and repair processes occurring at the cellular level.

The fundamental framework for understanding biological wear is the concept of allostatic load, which represents the cumulative physiological cost of chronic adaptation to unfavorable environmental conditions. While allostatic mechanisms enable short-term survival under stress, poor nutritional patterns, sleep deprivation, and persistent psychological strain generate a long-term metabolic burden.

At the subcellular level, this burden manifests as sustained hyperreactivity of stress regulatory systems, mitochondrial dysfunction, and chronic sterile low-grade inflammation (inflammaging). Within this framework, biological aging can be interpreted as the accumulation of unresolved repair deficits, a state in which the magnitude of externally or internally generated molecular damage persistently exceeds the efficiency of nocturnal regenerative processes and the endogenous antioxidant capacity of cells [196].

The overall synthesis of the presented evidence indicates that lifestyle is a primary determinant of biochemical and physiological integrity. Optimization of sleep hygiene, regulation of psychological stress, and stabilization of glucose-insulin metabolism represent not only preventive strategies but also active subcellular interventions influencing gene expression and enzymatic activity, including telomerase and PARP1.

Each behavioral choice modifies the intracellular environment, influencing whether cells maintain functional homeostasis or progress toward premature senescence. Consequently, understanding and consciously managing lifestyle-related biological signaling constitutes one of the most effective strategies for preserving biological longevity and structural integrity of the organism.

## 6. Research Perspectives

Because cellular stress represents a common denominator across numerous pathological conditions, a key unresolved issue is whether cellular stress pathways can be modulated therapeutically without disrupting essential adaptive signaling. Cellular stress is not inherently detrimental; rather, it serves as an essential biological signaling mechanism.

Short-term, controlled stress activates adaptive pathways that enhance cellular resilience and strengthen defense mechanisms. Pathology emerges when stress exposure becomes chronic and exceeds the compensatory capacity of protective systems. Modern biomedical research therefore does not aim to eliminate cellular stress entirely. Instead, the primary objective is to restore equilibrium between stress signaling and adaptive biological responses, promoting resilience rather than suppression of physiological regulatory processes.

In parallel with therapeutic research, the concept of utilizing cellular stress biomarkers for the diagnosis and monitoring of chronic diseases is rapidly developing [197,198,199]. Instead of focusing exclusively on organ-level manifestations, increasing attention is being directed toward molecular indicators of oxidative stress, ER stress, DNA damage, and mitochondrial dysfunction. This biomarker-based approach may enable earlier detection of chronic non-communicable diseases, more accurate risk stratification, and continuous monitoring of therapeutic efficacy. Such monitoring may be applied to both pharmacological interventions and lifestyle-based modulation strategies.

Contemporary biomedical science is gradually shifting its focus from symptomatic suppression toward reinforcement of endogenous cellular defense systems. Rather than asking how to completely eliminate stress signaling, researchers are increasingly investigating how to enhance cellular adaptive capacity. This perspective integrates biochemistry, medicine, and lifestyle science into a unified conceptual framework, establishing a new paradigm of health and disease. Within this paradigm, the cell is regarded as the fundamental unit of biological resilience and systemic health regulation.

## 7. Conclusions

Although stress is most commonly associated with psychological tension, its most profound and long-term effects occur at the level of individual cells. Within mitochondria, ER, and the nucleus, critical biological decisions are made regarding adaptation, repair, or progressive functional decline. Increasing evidence suggests that cellular stress represents a common underlying mechanism in nearly all chronic diseases.

Cells possess highly developed defense and maintenance systems, including mechanisms that neutralize ROS, repair DNA damage, and remove dysfunctional proteins and mitochondria. Pathology emerges when stress exposure becomes chronic and repair systems are unable to compensate for the rate of molecular damage. In such conditions, stress ceases to function as a warning signal and instead becomes a driving force of disease progression.

Understanding cellular stress fundamentally changes the conceptualization of health and disease. Diseases are no longer viewed solely as the result of damaged organs but rather as manifestations of long-term dysregulation at the cellular level. This perspective bridges laboratory science with everyday behavior, demonstrating that lifestyle, environment, and circadian rhythm leave molecular imprints long before clinical symptoms appear.

Cellular dysfunction often manifests as gradual, quantifiable changes in molecular and physiological markers. These changes represent sensitive indicators of lifestyle-related biological risk-systems that merit careful monitoring before critical thresholds are reached.

## Data Availability

No new data were created or analyzed in this study. Data sharing is not applicable to this article.

## Abbreviations

The following abbreviations are used in this manuscript:

ACTH	adrenocorticotropic hormone
AD	Alzheimer’s disease
AGEs	advanced glycation end-products
AMPK	5^′^AMP-activated protein kinase
AQP4	aquaporin-4
ATG	autophagy-related genes
ATM	ataxia-telangiectasia mutated
ATP	adenosine triphosphate
BDNF	brain-derived neurotrophic factor
CAT	catalase
CHOP	C/EBP homologous protein
CNS	central nervous system
CRH	corticotropin-releasing hormone
DAMPs	damage-associated molecular patterns
DDR	DNA damage response
ECS	endocannabinoid system
ER	endoplasmic reticulum
ERAD	endoplasmic reticulum-associated degradation
FFA	free fatty acid
GPx	glutathione peroxidase
GSH	glutathione
GSSG	glutathione disulfide
GR	glucocorticoid receptors
HPA	hypothalamic-pituitary-adrenal
IL	interleukin
LDL	low-density lipoprotein
mTOR	mammalian target of rapamycin
NADPH	nicotinamide adenine dinucleotide phosphate
NREM	non-rapid eye movement
Ox-LDL	oxidized low-density lipoprotein
PD	Parkinson’s disease
RAGE	receptor for AGEs
RNS	reactive nitrogen species
ROS	reactive oxygen species
SASP	senescence-associated secretory phenotype
SOD	superoxide dismutase
TNF-α	tumor necrosis factor α
UPR	unfolded protein response
